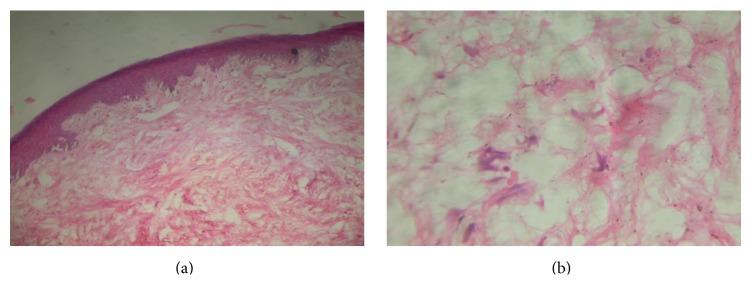# Giant Cell Fibroma in a Paediatric Patient: A Rare Case Report

**DOI:** 10.1155/2015/240374

**Published:** 2015-11-26

**Authors:** Veera Kishore Kumar Reddy, Naveen Kumar, Prashant Battepati, Lalitha Samyuktha, Swapna Priya Nanga

**Affiliations:** ^1^Department of Pedodontics and Preventive Dentistry, CKS Teja Institute of Dental Sciences, Tirupati 517501, India; ^2^Department of Pediatric Dentistry, SDM College of Dental Sciences and Hospital, Dharwad, Karnataka 580001, India; ^3^S. V. Medical College, Tirupati 517501, India

## Abstract

Giant cell fibroma is a form of fibrous tumour affecting the oral mucosa. Its occurrence is relatively rare in paediatric patients. Clinically it is presented as a painless, sessile, or pedunculated growth which is usually confused with other fibrous lesions like irritation fibromas. Here we are presenting a case where a seven-year-old male patient reported with a painless nodular growth in relation to lingual surface of 41 and 42. Considering the size and location of the lesion, excisional biopsy was performed and sent for histopathological analysis which confirmed the lesion as giant cell fibroma.

## 1. Introduction

Giant cell fibroma is a unique fibromucosal mass which clinically may resemble other fibrous growths like irritational fibroma. First reported by Weathers and Callihan in 1974, GCF if found predominantly in Caucasians under the age of 30 years with slight female predilection [1]. Its name is attributed to the characteristic histologic presentation, where there is presence of large multinucleated fibroblasts that tend to occur in close proximity to the overlying epithelium. It represent approximately 2–5% of all fibrous lesions submitted for biopsy and 0.4–1% of total biopsies, although greater percentages have also been presented [2]. Clinically giant cell fibroma is an asymptomatic sessile or pedunculated nodule, usually less than 1 cm in size. The surface of the mass often appears papillary. In about 60% of cases, the lesion is diagnosed during the first 3 decades of life and has slight female predilection. It is found more frequently on gingiva followed by tongue and buccal mucosa. Mandibular gingiva is affected twice as often as the maxillary gingiva [2].

Microscopically a giant cell fibroma is an uncapsulated mass of loose fibrous connective tissue that contains numerous characteristic large, plump, spindle shaped, and stellate fibroblasts, some of which are multinucleated. These cells are easily observed in the peripheral areas of the lesion [3]. The most accepted hypothesis for origin of GCF is as a response to trauma or to a recurrent chronic inflammation [4], characterized by functional changes in fibroblastic cells, while other cells would take over for collagen synthesis [5–7].

## 2. Case Report

**Figure 1:**
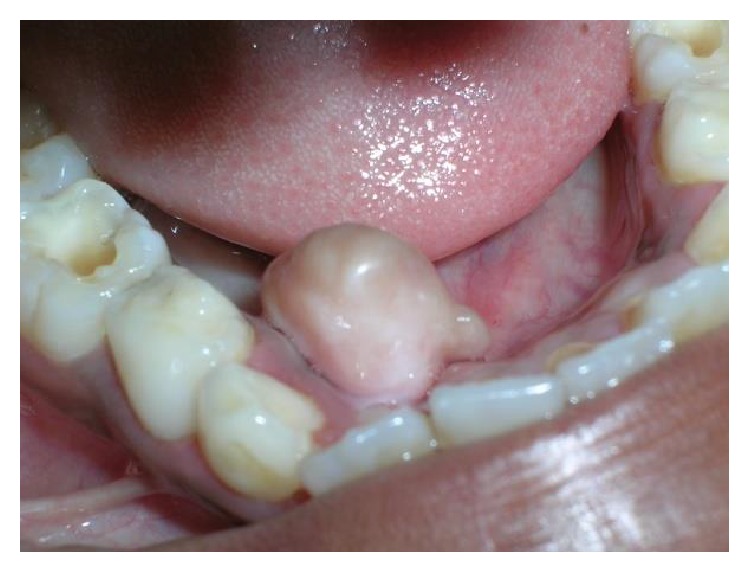


**Figure 2:**
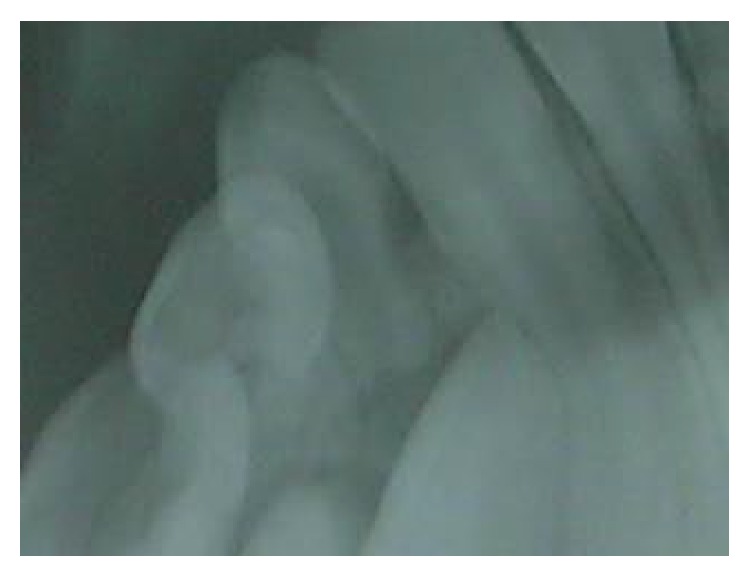


A 10-year-old male child reported to Department of Pediatric Dentistry with a complaint of a swelling below the tongue for the past 5 months. On detailed history recording, it was revealed that lesion is painless with no major changes in the size of the lesion since its appearance. On examination, a single pedunculated swelling which is firm and nontender on palpation is seen in relation to mandibular gingiva (lingual aspect) of 41, 42, 83, and 84 (Figure 1). Radiographic exam did not show any evidence of a lesion in hard tissue (Figure 2).

**Figure 3:**
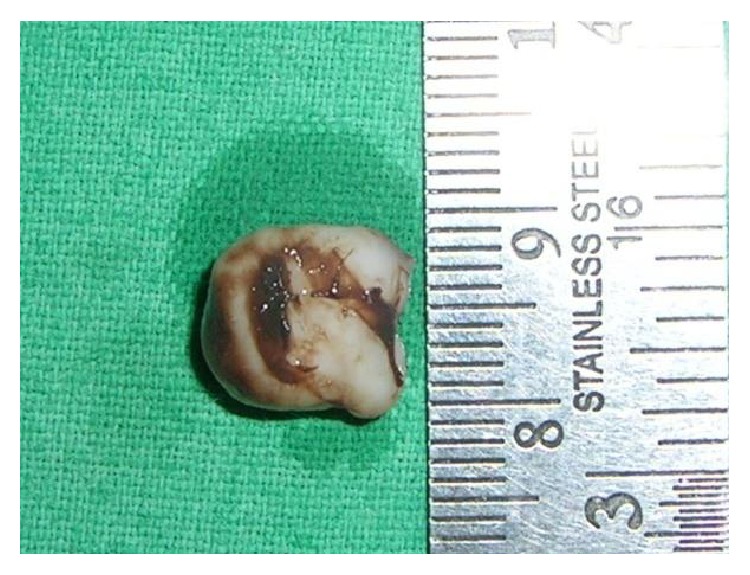


**Figure 4:**
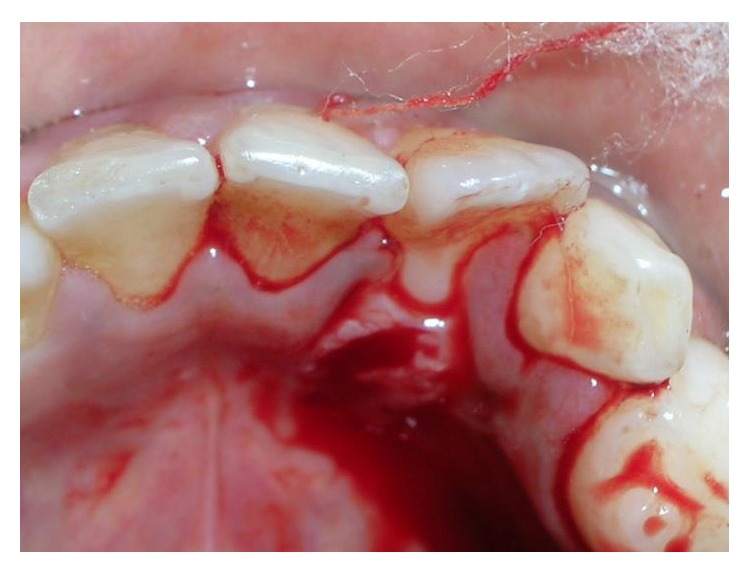


Based upon the clinical presentation of the lesion, the differential diagnosis was fibrous hyperplasia and fibroma was made. Considering the size and location of the lesion, excisional biopsy was performed under local infiltration anesthesia (Figures 3 and 4) and the specimen was sent for histopathological analysis.

Histological analysis of the excised lesion showed that bundles of connective tissue present a short course and interwoven fibres. Among them stellate shaped atypical fibroblasts and multinucleated giant cells are seen. Connective tissue is devoid of inflammatory cells. The overlying epithelium is of orthokeratinized stratified squamous type which is stretched out in few areas (Figures 5(a) and 5(b)).

The follow-up examination after a week confirmed uneventful healing. Patient was recalled after 3 months for routine follow-up to rule out any chances of recurrence.

## 3. Discussion

As evidenced in this case study, and in diagnosing lesions in general, both clinical and histologic features are important in determining a final diagnosis. In spite of similar histology, several distinctions can be made between a number of fibrous hyperplasias according to characteristics such as age distribution, gender predilection, location, and etiology.

GCF usually develops sometime in the first three decades of life; the lesions are usually <1 cm in diameter and are found more frequently on the tongue and gingiva. Mandibular gingiva is affected twice as often as the maxillary gingiva. In our case the size of the lesion is 9 mm found on lingual surface of mandibular gingiva in relation to 41, 42, 83, and 84 [3, 4, 8–11]; despite being rare, 5 percent to 15.5 percent of evaluated GCF had been found in children from birth to 10 years. Though few cases have been reported in both the genders no predilection towards any of the genders is witnessed [10–12].

Clinical and histological characteristics observed in this case are similar to others described in the literature, because of its clinical appearance of well grown delimited coloration similar to normal oral mucosa and papillary connective tissue, with short collagenous fibers and significant of giant cells [3, 4, 12, 13].

Considering clinical features, the differential diagnosis was fibroma and fibrous hyperplasia. In spite of being similar to a hyperplasia, the GCF presents age, sex, and race particularities which separate it from other entities [14, 15]. However, the histopathological analysis was required to conclude the diagnosis, so that some distinctive characteristics as presence of giant cells could be checked. The treatment for the lesion is surgical excision [3, 16].

## 4. Conclusion

Several fibrous hyperplastic lesions are similar both clinically and histologically, requiring biopsy for definitive diagnosis. Pedodontist should be familiar with the different types of fibrous hyperplasias they may encounter during patient treatment and should note such lesions for further evaluation by Oral and Maxillofacial Pathologist.

As demonstrated in this case study, GCFs may continue to proliferate until completely removed. GCFs can be treated by conservative surgical excision without subsequent recurrence. A case can be made for early recognition and treatment of lesions to minimize surgical intervention.

## Figures and Tables

**Figure 5:**